# Acoustic Detection of Forest Wood-Boring Insects Under Co-Infestations

**DOI:** 10.3390/insects16121241

**Published:** 2025-12-08

**Authors:** Qi Jiang, Yujie Liu, Yu Sun, Lili Ren, Youqing Luo

**Affiliations:** 1 Research Center for Natural Protected Areas Monitoring, Yunnan Institute of Forest Inventory and Planning, Kunming 650051, China; qiaoba78@126.com; 2 Laboratory for Pest Monitoring, Yinglin Branch Yunnan Institute of Forest Inventory and Planning, Kunming 650032, China; yujieliu@bjfu.edu.cn; 3 School of Cyber Science and Technology, Beihang University, Beijing 100191, China; sunyv@buaa.edu.cn; 4 Beijing Key Laboratory for Forest Pest Control, Beijing Forestry University, Beijing 100083, China; lily_ren@bjfu.edu.cn; 5 Sino-French Joint Laboratory for Invasive Forest Pests in Eurasia, Beijing Forestry University—French National Research Institute for Agriculture, Food and Environment (INRAE), Beijing 100083, China

**Keywords:** wood-boring insect, acoustic detection, feeding vibration signals, co-infestation scenarios, deep learning algorithms

## Abstract

Acoustic detection technology has emerged as a promising, non-destructive and continuous monitoring method for pest early detection at the single tree level. However, field application still encounters problems, especially under complex infestation scenarios, i.e., co-infestations by multiple pest species. This study aims to develop a novel acoustic-based recognition model for detecting forest wood-boring pests, specially designed to enhance monitoring accuracy under complex infestation scenarios. We collected feeding vibration signals from four wood-boring pests: *Semanotus bifasciatus*, *Phloeosinus aubei*, *Agrilus planipennis*, and *Streltzoviella insularis*. Three infestation scenarios were designed: single-species, co-infestation without mixed signals, and co-infestation with mixed signals. Three machine learning (ML) models (Random Forest, Support Vector Machine, and Artificial Neural Network) based on seven acoustic feature variables, and three deep learning (DL) models (AlexNet, ResNet, and VGG) using spectrograms were employed to classify the signals. Results showed that ML models achieved perfect accuracy (OA: 100%, Kappa: 1) in single-species scenarios but declined significantly under co-infestation scenarios with mixed signals. In contrast, DL models, particularly ResNet, maintained high accuracy (OA: 85.0–88.75%) and effectively discriminated mixed signals. In conclusion, this study demonstrates the superiority of spectrogram-based DL models for acoustic detection under complex infestation scenarios and provides a foundation for developing a general, real-time detection model for integrated pest management in forest ecosystems.

## Simple Summary

Forest trees are increasingly threatened by wood-boring insects that live hidden inside trunks, making early detection difficult until the trees are severely damaged or dead. This study introduces an innovative acoustic detection technology to early detect insects attack and identify insect species. We recorded the sounds of four different wood-boring insect species, designed three infestation scenarios, and tested different computer models to identify them, especially in complex situations where multiple species infest the same tree and their sounds mix. While machine learning models performed perfectly in single-species scenarios, their accuracy dropped when mixed signals were present. In contrast, deep learning models maintained high accuracy, successfully identifying up to 88.75% of the pests even when their sounds overlapped. This research provides a foundation for developing smart, real-time detection devices that can help protect forests and valuable trees by enabling early and accurate pest detection.

## 1. Introduction

Forest wood-boring pests are considered the predominant biotic threat, causing considerable damage to global forest ecosystems [1,2]. Generally, larvae or adults of these pests burrow under the trunk xylem or phloem and bore dense tunnels that block the plants’ nutrients and water transport, ultimately causing the infested trees to wither and die. Unlike the obvious symptoms (e.g., crown defoliation and dieback) in the case of forest defoliators, wood-boring pests have a hidden life history (during the larval growth stages) and delayed damage symptoms. These characteristics cause the attacked trees that are often only detected in the middle or late stages of infestation, severely limiting the effectiveness of control measures. This delayed detection facilitates rapid and widespread infestations at later stages, leading to large-scale tree death and posing a serious threat to urban greening, especially the conservation of notable and ancient trees [3,4,5,6]. Consequently, there is an urgent need for tools that can rapidly detect and accurately identify wood-boring pest damage during the early stages of infestation.

Currently, there are several methods to detect wood-boring pests: (1) visual inspection; (2) insect pheromone traps; (3) X-ray image analysis; (4) acoustic detection and (5) remote sensing technology [2,7,8,9]. Among these methods, acoustic detection has gained increasing attention due to its advantages of being non-destructive and offering long-term monitoring and high accuracy, especially for detecting early-stage pest infestations (larval growth stages) in single trees [6,8,10]. The principle of acoustic detection of wood-boring pests is as follows: as insect larvae move and feed beneath the trunk xylem or phloem, they stress and snap wood fibers. This activity generates vibration signals, which can be captured by highly sensitive sensors. These signals, characterized by trains of brief, high-amplitude pulses and distinctive spectral patterns, can be readily distinguished from background noise and other signals [8,11,12,13,14]. Therefore, they can be analyzed to determine the presence and species of the pests, as well as other infestation-related details [8].

The advancements in bioacoustic recognition technology and the evolution of acoustic detection instruments have greatly broadened the application of acoustic detection in monitoring early-stage pest infestations. Many studies have been reported using acoustic detection for identifying insect species, estimating population, mapping distributions and analyzing activity patterns [8,15]. For example, Sun et al. (2020) [16] designed the InsectFrames of a lightweight CNN to automatically identify larvae vibration signals of Semanotus bifasciatus (Coleoptera: Cerambycidae) and Eucryptorrhynchus brandti (Coleoptera: Curculionidae), with an accuracy of 95.83%. By analyzing the temporal variation patterns of the feeding signals, Jiang et al. (2022) [10] determined that the best detection time window for early detecting S. bifasciatus and established models for predicting larval instars and population size.

Although existing studies have demonstrated the feasibility of acoustic detection technology for early monitoring of pest infestations, field practical application still faces numerous challenges that require further exploration. One of the key challenges is the accurate identification of wood-boring pest species under complex infestation scenarios, including single-species infestations and co-infestations by multiple species. In the field environment, host trees are often attacked by multiple wood-boring pest species, resulting in complex infestation scenarios [17,18]. This phenomenon means that acoustic detection sensors deployed in the field may capture signals from either single-pest species or multiple-pest species within the same tree trunk, or even mixed signals during multi-species co-infestation. The performance of recognition algorithms can exhibit substantial fluctuations in accuracy across different infestation scenarios, particularly when distinguishing similar signals generated from different pests.

At present, pest acoustic detection models are primarily categorized into two types: the first type is machine learning models based on acoustic feature variables; the second type is deep learning models based on spectrograms. The former models can achieve stable training with limited labeled data, and their feature parameters possess explicit biological acoustic interpretability. The latter employs an end-to-end automatic feature extraction approach, effectively avoiding biases that may arise from manual feature design, and demonstrates significant performance advantages when handling large-scale data. For example, Luo et al. (2011) [19] extracted 12-dimensional Mel-Frequency Cepstral Coefficients (MFCCs) from the acoustic signals of four bark beetle species and achieved an identification accuracy of over 90% using a BP neural network for training and detection. Jiang et al. (2024) [20] input the acoustic spectrograms of Agrilus planipennis Fairmaire (Coleoptera: Buprestidae) and Holcocerus insularis (Lepidoptera: Cossidae) into the Residual Mixed Domain Attention Module Network (RMAMNet), with a classification accuracy of 95.34%. However, there have been no reports of simultaneous application of these two models in complex situations, especially in mixed infestation situations of wood-boring pests. Therefore, it remains to be further explored and verified which model performs better in complex situations.

**Figure 1:**
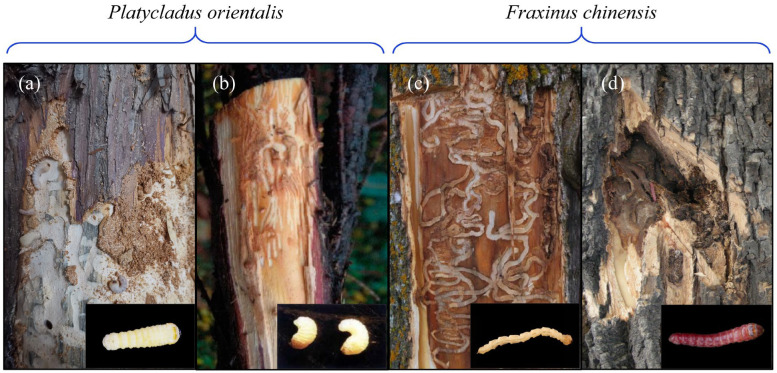
Larvae and infestation symptoms of four wood-boring pests: (**a**) *S. bifasciatus*; (**b**) *P. aubei*; (**c**) *A. planipennis*; (**d**) *S. insularis*.

The primary objective of this study was to develop an acoustic detection model capable of accurately identifying forest wood-boring pests in complex infestation scenarios. We selected four wood-boring pest species as experimental subjects: *Semanotus bifasciatus* Motschulsky (Coleoptera: Cerambycidae), *Phloeosinus aubei* Perris (Coleoptera: Scolytidae), *Agrilus planipennis* Fairmaire (Coleoptera: Buprestidae) and *Streltzoviella insularis* Staudinger (Lepidoptera: Cossidae) (Figure 1). Field investigations revealed that in some cases, *Platycladus orientalis* was simultaneously infested by *S. bifasciatus* and *P. aubei*, and *Fraxinus chinensis* was co-infested by *A. planipennis* and *S. insularis*. The experiments were conducted through the following steps: (1) compare and elucidate time-frequency characteristics of the feeding vibration signals from these four pest species; (2) establish datasets separately for three distinct infestation scenarios: single-species infestation, co-infestation without mixed signals, and co-infestation with mixed signals; (3) employ three machine learning algorithms based on acoustic feature variables and three deep learning algorithms based on acoustic spectrograms to develop models. This research is expected to provide valuable insights for the development of a general acoustic recognition model for wood-boring pests in forest ecosystems and contribute to the improvement of integrated pest management strategies.

## 2. Materials and Methods

### 2.1. Experimental Logs

In 2021, experimental logs (length 50 cm) of *P. orientalis* and *F. chinensis* were collected from Beijing forest farms. The types of logs collected for this study included: (1) *P. orientalis* logs infested exclusively by either *S. bifasciatus* or *P. aubei* larvae; (2) *F. chinensis* logs infested exclusively by either *A. planipennis* or *S. insularis* larvae; and (3) healthy, non-infested logs of both *P. aubei* and *F. chinensis*. Three replicates were collected per infestation type, with single controls per species. All logs underwent rigorous visual verification of target larval presence in infested samples and absence in controls.

### 2.2. Acoustic Detection Instrument

In this experiment, the feeding vibration signals of wood-boring pests were recorded and collected using an improved acoustic detection instrument based on the AED-2010L (Acoustical Emission Consulting, Inc., Fair Oaks, CA, USA) system. The original system comprises three modules: (i) a magnetic attachment (Model DMH-30; Acoustical Emission Consulting, Inc.) for connecting the waveguide screw; (ii) a sensor-preamplifier module (Model SP-1L; Acoustical Emission Consulting, Inc.), and (iii) an amplifier (AED-2010L; Acoustical Emission Consulting, Inc.) which connected to a digital audio recorder.

**Figure 2:**
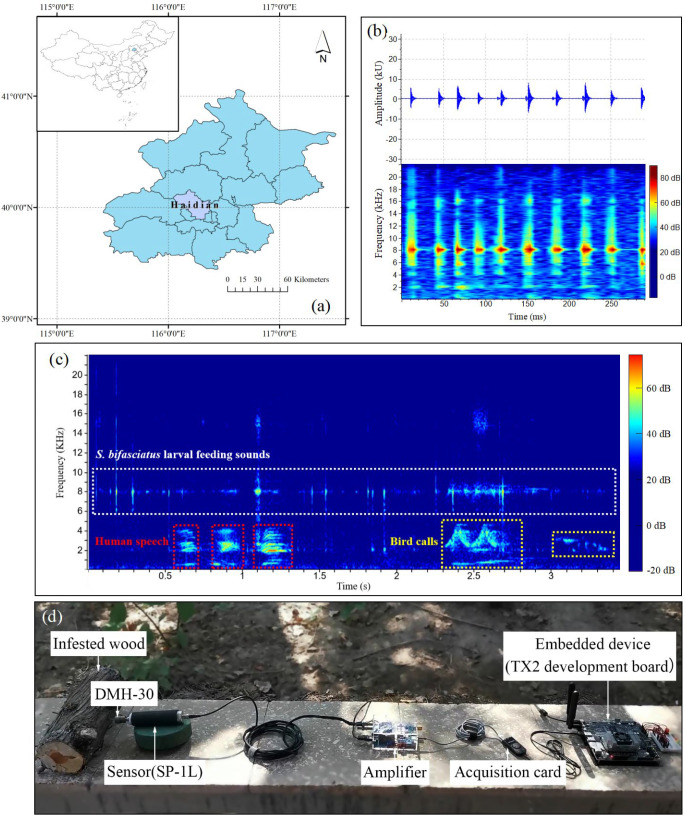
Study location, environmental noise type and acoustic detection instrument. (**a**) Experiment location. (**b**) An example of an oscillogram and spectrogram of the larval feeding vibration signals. (**c**) An example of an environmental noise spectrogram, including human speech and bird calls. (**d**) The improved instrument used for vibration signal recording and collecting.

To enhance signal clarity and reduce noise interference, the whole instrument used in this study was improved in the laboratory at Beihang University. The Model DMH-30 and Model SP-1L probe were retained (Figure 2d) while integrating a self-developed amplifier circuit board into the sensor. The original AED-2010 amplifier was replaced with an amplifier (40× magnification), a data acquisition card, and an embedded device (TX2 development board) (Figure 2d). In the future, the trained general recognition model will be deployed on the embedded device to achieve offline, real-time detection of wood-boring pest signals in the field.

### 2.3. Feeding Vibration Signals Recording

All signal data were recorded outdoors (far from the blocks) in Haidian District, Beijing (Figure 2a). The environmental noise mainly included human speech and bird calls (Figure 2c). In preparing each log for recording, a 1.6-mm-diameter, 76-mm-long signal waveguide screw was inserted near larval feeding active sites in infested logs or at the center of non-infested logs. The feeding vibration signals of *S. bifasciatus* larvae and *P. aubei* larvae were recorded in mid-April 2021, whereas those of *A. planipennis* larvae and *S. insularis* larvae were recorded in early June. These recordings were carried out continuously for 7 days, with signal collection scheduled daily between 13:00 and 18:00, corresponding to the peak period of larval activities [8,10,21]. During each recording, non-infested logs were recorded first for 10 min (the sounds designated as “background noise”), followed by a 30-min recording of each infested log. The sampling rate and bit depth were set to 44.1 kHz and 32 bits, respectively.

### 2.4. Signals Processing and Features Extraction

Firstly, the recordings were prescreened using Adobe Audition CC 2018 software (Adobe, San Jose, CA, USA) to locate and select target pest feeding vibration signals. Specifically, 100 discrete audio segments (30 ms each) containing a single feeding vibration pulse were extracted from the recordings of each pest species. Additionally, 100 audio segments (30 ms each) of background noise were selected from the recordings of non-infested *P. orientalis* log and *F. americana* logs, respectively. Then, using the mix-and-paste function in Adobe Audition, the feeding vibration pulses of two different pests were combined in a 1:1 ratio to simulate the simultaneous presence of both pests. This process generated two types of mixed signals: (1) *S. bifasciatus* + *P. aubei* mixed signals, and (2) *A. planipennis* + *S. insularis* mixed signals, with 100 audio segments (30 ms each) for each mixed signal type. Finally, a set of feature variables and spectrograms was extracted and generated from these segments using Raven Pro 1.6 software (The Cornell Lab of Ornithology, Ithaca, NY, USA).

In Raven Pro, the measurement chooser enables users to select any combination of measurements [22]. For target detection, we used two detectors, namely an amplitude detector and a band-limited energy detector, to configure the necessary parameters. Seven feature variables were measured from the oscillograms and spectrograms (Figure 2b) (Table 1): max amplitude (Max Amp), min amplitude (Min Amp), RMS amplitude (RMS Amp), average power density (Avg PD), energy (Energy), peak power density (Peak PD), and peak frequency (Peak Freq). The spectrograms were generated using the short-time Fourier transform (STFT) with Hann windows of 512 samples. The window overlap was set to 50%, and the hop size was 256 samples. Additional parameters included a frequency grid spacing of 86.1 Hz and a filter bandwidth of 124 Hz at the 3 dB level.

### 2.5. Datasets Construction Under Different Damage Scenarios

In this study, two datasets were constructed based on the corresponding relationship between wood-boring pests and host tree species (Table 2): *P. orientalis* dataset (Dataset 1, D1) and *F. chinensis* dataset (Dataset 2, D2). The D1 included the following signal categories: (1) the feeding vibration signals of *S. bifasciatus* larvae and *P. aubei* larvae; (2) mixed signals (M1) generated by combining *S. bifasciatus* larvae and *P. aubei* larvae; and (3) background noise (B1) recorded from non-infested *P. orientalis* log. The D2 included: (4) the feeding vibration signals of *A. planipennis* larvae and *S. insularis* larvae; (5) mixed signals (M2) generated by combining *A. planipennis* larvae and *S. insularis* larvae; and (6) background noise (B2) recorded from a non-infested *F. chinensis* log.

Depending on different damage scenarios, data subsets (Table 3) from D1 and D2 were constructed for model establishment. For the single-species infestation scenario, the subset comprised two categories: feeding vibration signals from a single-pest species and background noise from the corresponding dataset. For the scenario of co-infestation by two pest species, the subset included three categories: feeding vibration signals from two pest species and background noise from the same Dataset. Furthermore, the category of mixed signals was added to the above subset to simulate the scenario where two pest species simultaneously damage the same host tree.

During model establishment, seven feature variables and spectrograms were extracted and generated from all audio segments (.wav) within each dataset using Raven Pro 1.6. For each signal category, 80% of the samples (80 segments per category) were randomly selected as the training set, while the remaining 20% (20 segments per category) were used as the test set for model accuracy evaluation.

### 2.6. Model Establishment and Accuracy Evaluation

#### 2.6.1. Machine Learning Models

**Figure 3:**
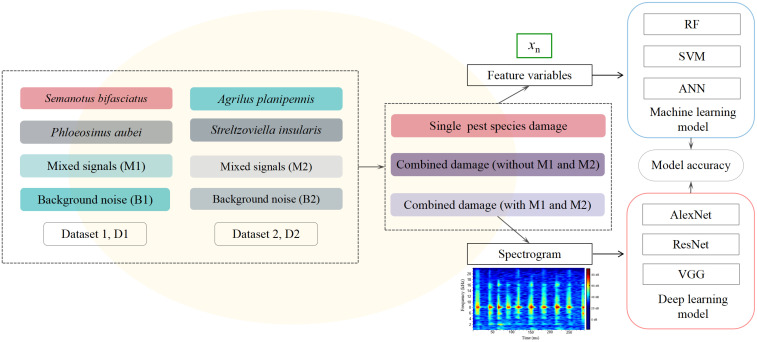
Datasets construction and modeling process under different damage scenarios.

Non-parametric machine learning (ML) algorithms with internal cross-validation have been extensively applied for developing classification models in bioacoustic research. Among these, Random Forest (RF), Support Vector Machine (SVM), and Artificial Neural Network (ANN) are particularly prominent due to their superior performance [23,24,25]. In this study, the R software (R Studio 4.2; R studio Inc., Boston, MA, USA) packages “randomForest” (RF), “e1071” (SVM), and “nnet” (ANN) were employed to establish three machine learning models. Seven feature variables extracted from the audio segments were taken as inputs for all three algorithms (Figure 3).

#### 2.6.2. Deep Learning Models

Three classic deep learning (DL) models (AlexNet, ResNet, and VGG) were established for the classification of wood-boring pest species, using spectrograms generated from audio segments as input (Figure 3).

The AlexNet, proposed by Alex Krizhevsky et al. [26] is a pioneering convolutional neural network (CNN) for image classification. This architecture (model layer structure) consists of eight layers: five convolutional (sliding-feature extraction) layers, interleaved with max-pooling layers, followed by three fully connected layers. The max-pooling layers reduce the dimensions of the feature maps while preserving spatial invariance. Key innovations include the ReLU (Rectified Linear Unit) activation function to mitigate vanishing gradients and dropout regularization to prevent overfitting. Local response normalization (LRN) further enhances generalization. The VGG, developed by the Visual Geometry Group at Oxford University [27], is a significant milestone in CNN design. Unlike AlexNet-inspired works focusing on small kernels (feature-detecting filter) or multi-scale processing, VGG increases depth with 3 × 3 filters, balancing model capacity and computational efficiency. The ResNet, introduced by He et al. (2016) [28] at Microsoft Research, achieved first place in the ImageNet Large-Scale Visual Recognition Challenge (ILSVRC) in 2015 for both image classification and object detection tasks. ResNet addressed the degradation problem with residual learning and skip connections. This architecture enables stable training of ultra-deep networks (up to 152 layers) by preserving gradient flow, significantly enhancing the extraction of multi-level latent features from complex data like spectrograms.

The training and testing process of three DL models was conducted on a Windows 10 64-bit operating system, using the TensorFlow open-source deep learning framework. The hardware configuration included an AMD R7 6800H CPU (4.40 GHz) paired with an NVIDIA GeForce RTX 3060 GPU (8 GB VRAM). Model implementation was performed using Python 3.9.7 and PyTorch 1.9.0, with GPU acceleration enabled via CUDA 11.6. The training process was configured with the following parameters: 300 training epochs, an initial learning rate of 0.0001, and a mini-batch size of 16 samples. Additionally, the momentum (optimization parameter for faster convergence) and weight decay (regularization to prevent overfitting) were set to 0.9 and 0.0005, respectively. To mitigate overfitting, an early stopping mechanism was implemented during the training.

#### 2.6.3. Model Accuracy Assessment

The accuracy of ML models and DL models was evaluated using the confusion matrix, Overall accuracy (OA), and Cohen’s Kappa coefficient [29]. Cohen’s Kappa coefficient is a statistical measure used to assess the consistency between predicted and actual outcomes, reflecting the reliability of a classification model. It typically ranges from −1 to 1, with higher values indicating better consistency in the model’s classification results and greater model reliability. In addition, evaluation parameters based on the loss function served as critical indicators of model convergence and potential overfitting [30].

## 3. Results

### 3.1. Time and Frequency Domain Characteristics of Feeding Vibration Signal

**Figure 4:**
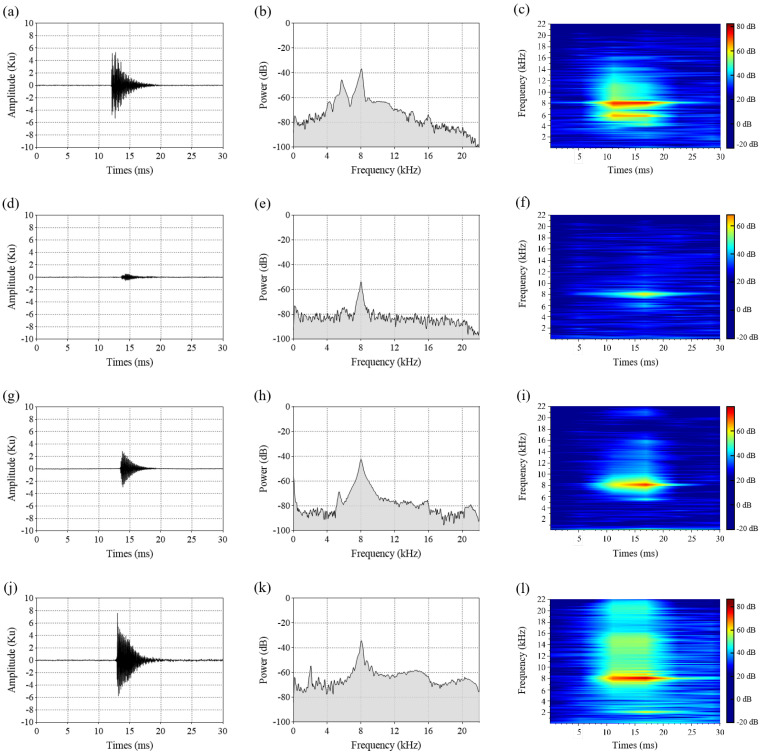
Oscillograms (left column), average power spectrums (center column), and spectrograms (right column) depicting a single feeding pulse for larvae of four pest species: *S. bifasciatus* (**a**–**c**), *P. aubei* (**d**–**f**), *A. planipennis* (**g**–**i**), and *S. insularis* (**j**–**l**).

The feeding activity of wood-boring pest larvae within tree trunks generates trains (groups) of 3 to 30 ms pulses [8,10,11,31]. The feeding vibration signals of four pest species larvae consisted of discrete pulses, each pulse lasting less than 30 ms, with irregular intervals between consecutive pulses. Oscillograms of a single feeding pulse from these four pest species (Figure 4a,d,g,j) revealed similar waveform characteristics: a fast-rising front followed by a ‘tail’ with a time decay. The oscillograms were similar to those observed in previously published research [11,15,32].

Compared with the feeding pulse amplitudes of *P. aubei* larvae and *A. planipennis* larvae, those of *S. bifasciatus* larvae and *S. insularis* larvae were higher (Figure 4a,d,g,j), indicating greater sound intensity in the latter species. The average power spectrums and spectrograms showed that the feeding pulses of these pests exhibited a broad frequency bandwidth (0–20 kHz) (Figure 4b,e,h,k), with high power concentrated between 7 kHz and 9 kHz (Figure 4c,f,i,l). The peak frequency reached approximately 8 kHz.

### 3.2. Classification Accuracy of Machine Learning Models Based on Seven Feature Variables

**Figure 5:**
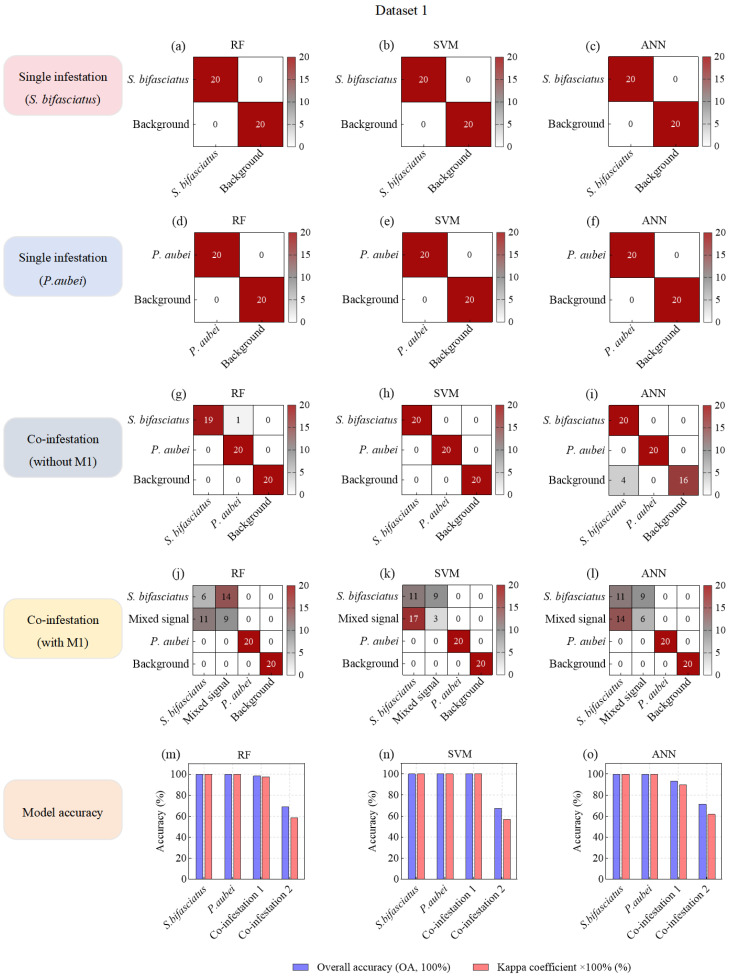
Confusion matrices of machine learning models based on acoustic feature variables for species classification of *P. orientalis* wood-boring pests.

**Figure 6:**
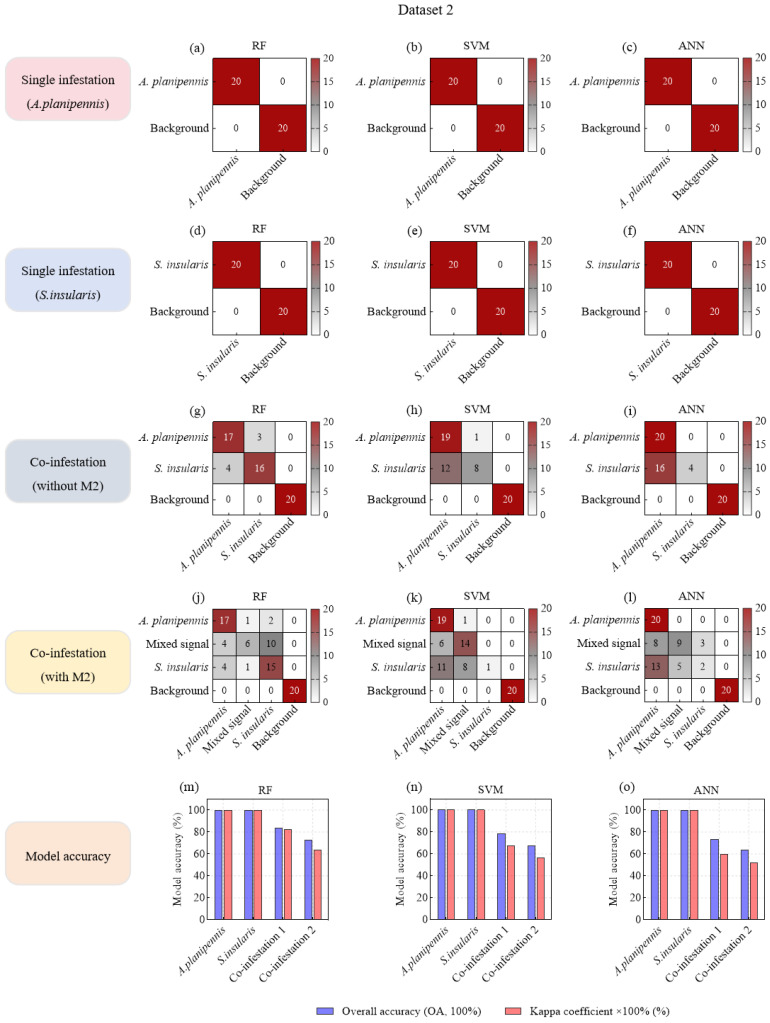
Confusion matrices of machine learning models based on acoustic feature variables for species classification of *F. chinensis* wood-boring pests.

In the single-species damage scenario, all three ML models (RF, SVM, and ANN) achieved perfect accuracy (OA—100%, Kappa—1) in identifying specific pest species from background noise in both D1 and D2 (Figure 5a–f; Figure 6a–f). Moreover, under co-infestation scenarios and when mixed signals were added to datasets, background noise was still completely distinguished from larval feeding vibration signals (Figure 5g–l; Figure 6g–l).

In the co-infestation scenarios without mixed signals, the OAs of the three ML models decreased slightly. Notably, the model recognition accuracy in D1 (OA: 93.33–100.00%; Kappa: 0.9–1) was generally higher than that in D2 (OA: 73.33–83.33%, Kappa: 0.6–0.825) (Figure 5m–o; Figure 6m–o). This discrepancy might be attributed to the more distinct differentiation in feeding vibration signals between *S. bifasciatus* and *P. aubei*, compared to the relatively similar signals produced by *A. planipennis* and *S. insularis*. Particularly, in SVM and ANN models, a large number of S. insularis signals (4–16 samples) were misclassified as *A. planipennis* signals (Figure 6g–i).

In the co-infestation scenarios with mixed signals, the recognition accuracy of all three models in D1 and D2 significantly decreased. Specifically, in D1, the OAs dropped from 93.33–100.00% (Kappa: 0.9–1) to 67.5–71.25% (Kappa: 0.5667–0.6167), representing a nearly 30% decrease (Figure 5m–o). In D2, the OAs dropped from 73.33–83.33% (Kappa: 0.6–0.825) to 63.75–72.5% (Kappa: 0.5167–0.6333), representing a nearly 10% decrease (Figure 6m–o). This accuracy decline could be attributed to the overlap of two types of feeding vibration signals, where stronger signals masked weaker ones, resulting in mixed signals. These mixed signals exhibited characteristics similar to the stronger signals, leading to misclassification and reduced model accuracy. For example, in models built with D1, a large number of mixed signals (M1) were misclassified as S. bifasciatus signals (Figure 5j–l), significantly lowering the OA and Kappa coefficient. Although this misclassification did not affect the model’s ability to detect and identify the *S. bifasciatus* signals, it resulted in the *P. aubei* signals being missed. In Dataset 2, the feature similarity between some *A. planipennis* signals and *S. insularis* signals caused the mixed signals (M2) to be misclassified as either *A. planipennis* signals (4–8 samples) or as *S. insularis* signals (3–10 samples) (Figure 6j–l).

In summary, under the single-species infestation scenarios, the three ML models (RF, SVM, and ANN) based on seven feature variables demonstrated robust performance in accurately identifying target pest signals. In the co-infestation scenarios without mixed signals, although the overall classification accuracy of these models declined, the RF models still maintained relatively high accuracy in distinguishing between the feeding vibration signals of two pest species. However, when two pest species fed simultaneously and generated mixed signals, the OAs of all three models dropped below 75%, struggling to detect the presence of the pest with weaker signals. Therefore, it was necessary to introduce higher-performance models to mitigate this issue of miss detection and to enhance the classification accuracy in co-infestation scenarios involving mixed signals.

### 3.3. Classification Accuracy of Deep Learning Models Based on Spectrograms

**Figure 7:**
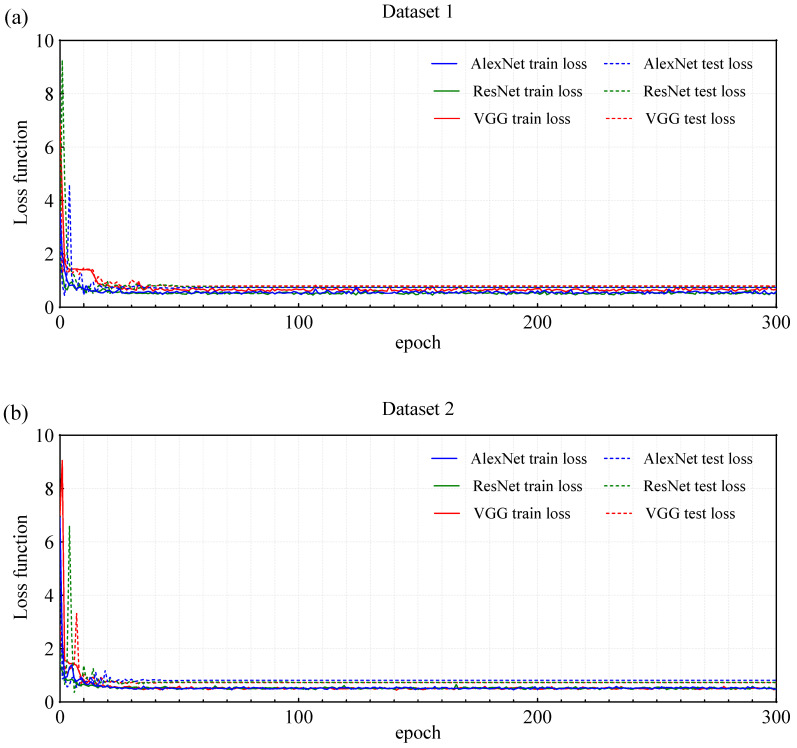
Loss function curve of three deep learning models trained by Dataset 1 and Dataset 2.

To address the issue of miss detection in co-infestation scenarios with mixed signals, this study further introduced three DL models (AlexNet, ResNet, and VGG) based on acoustic spectrograms, aiming to improve classification accuracy. As shown in Figure 7, the loss function curves of the three deep learning models (AlexNet, ResNet, and VGG) trained on Dataset 1 and Dataset 2 demonstrated no significant overfitting. In both Datasets, the training loss and test loss decreased steadily and remained close. Specifically, in Dataset 1, the loss values of the three models stabilized when the epoch reached 50 (Figure 7a), while in Dataset 2, the loss values stabilized at epoch 30 (Figure 7b). The models generally exhibited good generalization ability. These observations indicated that the models generally possess good generalization ability.

**Figure 8:**
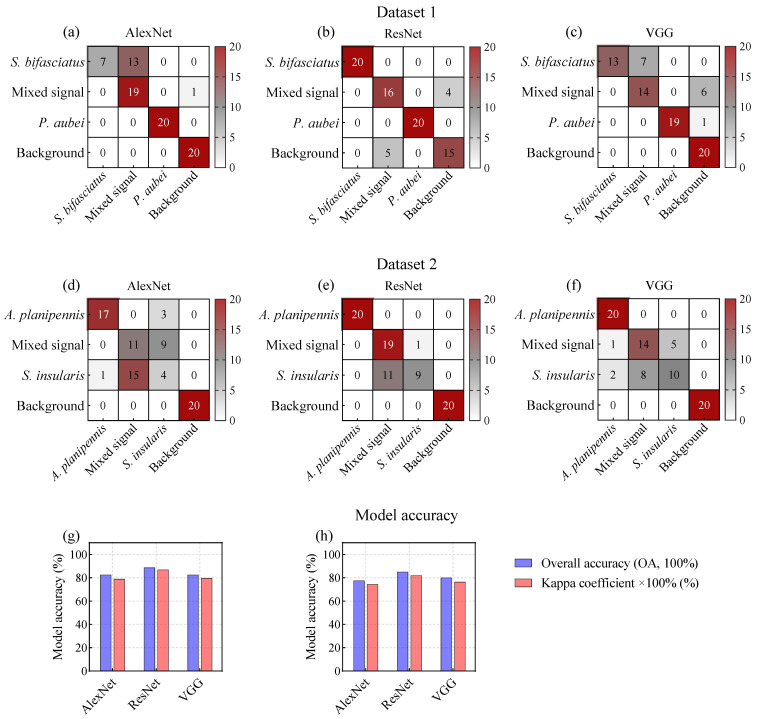
Confusion matrices of deep learning models based on spectrograms for species classification of *P. orientalis* and *F. chinensis* wood-boring pests.

The results demonstrated a significant improvement in model accuracy. In D1, the OAs increased from 68.75–71.25% to 82.5–88.75%, while the Kappa coefficients improved from 0.5667–0.6167 to 0.7883–0.8691 (Figure 8g). Similarly, in D2, the OAs increased from 63.75–72.5% to 77.5–85.0%, and the Kappa coefficients improved from 0.5167–0.6333 to 0.7444–0.8199 (Figure 8h).

This improvement highlighted the effectiveness of three DL models in enhancing model performance under co-infestation scenarios involving mixed signals, such as the correct identification of 14–19 mixed-signal samples in D1 (Figure 8a–c) and 11–19 samples in D2 (Figure 8d–f). Furthermore, while effectively reducing the interference from mixed signals, the models maintained high accuracy in identifying specific pest signals. Notably, ResNet outperformed AlexNet and VGG in both D1 and D2, rendering it a preferable choice for the detection of wood-boring pest signals.

## 4. Discussion

### 4.1. Factors Affecting Model Accuracy

The characteristics of insect acoustic signals are strongly influenced by species, growth stage, size, and the mechanisms and locations of sound production [8]. For wood-boring pests, signal production is closely linked to larval feeding activity: as larvae bore within the host tree trunk, fracturing wood fibers generates mechanical vibrations, producing feeding vibrations [8,10]. Consequently, factors such as larval species, feeding organ morphology, location within the wood (e.g., phloem vs. xylem), and host wood hardness significantly shape the characteristics of these feeding vibrations.

Comparisons of model accuracy between D1 and D2 revealed generally lower classification accuracy in D2. This reduction in accuracy may stem from two primary factors: interference from mixed signals and the misclassification of signal samples. For instance, some *S. insularis* feeding signals were misclassified as *A. planipennis* signals. This is likely attributable to the dispersed and uneven feeding sites of *S. insularis* larvae, which resulted in significant variations in the time-frequency characteristics of their recorded feeding signals. Consequently, when *A. planipennis* and *S. insularis* larvae concurrently bored and fed within the same wood location, the feeding signals they generated exhibited highly similar characteristics, thereby increasing the likelihood of model misclassification. Additionally, in the future, we will revisit and analyze signals containing trains of pulses rather than 30 ms periods. The differences in temporal patterns between consecutive pulses generated by different pest species, especially those from different genera, are likely to provide more distinctive and robust features for classification algorithms, potentially mitigating the interference of mixed signals.

Furthermore, the representational capacity and comprehensiveness of features used for model training significantly impact model performance [33,34,35]. In datasets where mixed signals constituted an interference category, the accuracy of three DL models based on spectrograms was significantly higher than that of three ML models based on feature variables. This performance gap likely stems from the fact that the acoustic feature variables were derived from a dimensionality reduction process applied to the raw feeding vibration signals, inevitably leading to the loss of certain signal characteristics. In contrast, spectrograms preserve a more comprehensive spectral characteristics of signals, thereby providing richer input data for model training and enabling more effective learning of complex patterns.

Finally, in practical applications of acoustic technology for detecting wood-boring pests, sensor placement is critical to detection accuracy because of acoustic attenuation during signal propagation. This attenuation—resulting from geometric spreading, medium absorption, and interface interactions (e.g., reflection and scattering)—weakens signal amplitude and energy with distance [36,37]. When signal propagation distances exceed a certain threshold, feature degradation and signal distortion reduce model recognition accuracy. Subsequent research will therefore analyze attenuation patterns of pest feeding vibrations propagating through trunks, establishing optimal sensor deployment heights to enhance detection precision.

### 4.2. General Recognition Model and Acoustic Database for Wood-Boring Pests

For field monitoring of individual trees, particularly notable and ancient trees, real-time acoustic monitoring with instant result feedback is crucial to reduce labor and monitoring costs. Such a system enables forestry workers to promptly analyse pest dynamics and implement timely management measures. Developing a comprehensive acoustic database for wood-boring pests and a general recognition model is essential for constructing this monitoring system.

**Figure 9:**
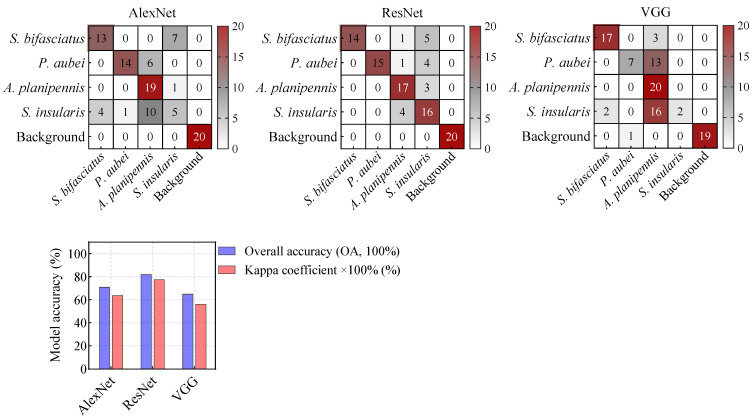
Confusion matrices of deep learning models based on spectrograms for species.

This study assessed the feasibility of developing a general recognition model for wood-boring by merging D1 and D2 to train three DL models. The results showed that the ResNet model could effectively distinguish the feeding signals of four pest species, achieving an OA of 82.00% and a Kappa coefficient of 0.775 (Figure 9). Notably, the model maintained robust accuracy without significant degradation compared to the Figure 7 results, indicating strong potential for scalable generalization.

Moreover, dataset size critically influences algorithm selection and model performance. Traditional ML models generally outperform DL models on small datasets, as DL models are prone to overfitting with limited samples [38,39,40]. While ML model performance improves with sample size growth, it eventually saturates due to limited capacity for complex data patterns [41,42]. In contrast, DL model performance improves near-linearly with increasing data volume, enabled by automatic hierarchical feature extraction and mitigating overfitting through large-scale data training [27,40,43,44,45]. Consequently, as training data diversity and volume expand, we prefer to employ lightweight DL models to develop a general recognition model for wood-boring pests.

### 4.3. Practical Application of Acoustic Detection in Forest Environments

Currently, the widespread application of acoustic detection technology is mainly limited by two factors: (1) labor-intensive, multi-stage audio data process workflows (acquisition, export, and post-processing), and (2) insufficient model robustness against diverse background noises commonly present in forest environments [6,46,47,48]. To address these challenges, we propose solutions from both hardware and algorithmic perspectives.

**Figure 10:**
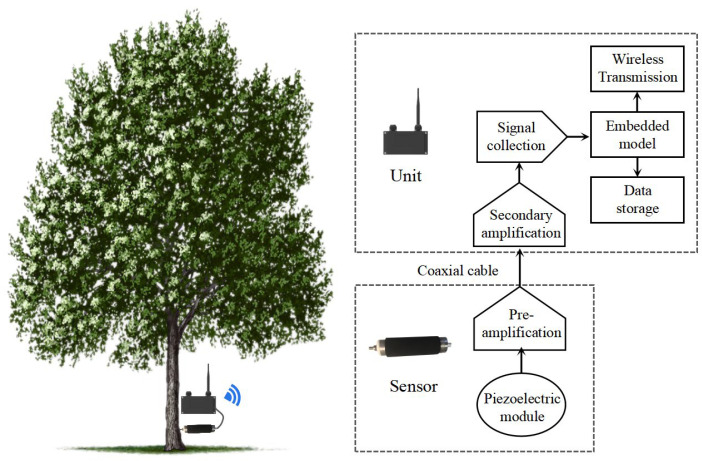
The architecture of an acoustic instrument for detecting wood-boring pests in fields.

To enable scalable, long-term remote monitoring, we propose developing a compact, low-power and IoT-oriented detection device for field deployment, as shown in Figure 10. This device integrates signal amplification, data acquisition, and a lightweight recognition model into a single embedded system, enabling real-time analysis of acoustic signals directly at the monitoring site, eliminating the need for manual data collection and offline processing.

Furthermore, studies have demonstrated the considerable potential of fiber-optic acoustic sensors in the early detection of wood-boring pests and in monitoring their spatial distribution [49,50,51]. Therefore, in subsequent research, we plan to introduce distributed acoustic sensors (based on fiber-optic acoustic sensors) and systematically compare them with the pointwise acoustic sensor (AED-2010L) employed in this study. This comparison aims to thoroughly evaluate its performance and application potential for the early detection, species identification, and spatial distribution mapping of wood-boring pests. This work will also provide experimental support at the sensor level for the development of more efficient acoustic detection devices for such pests.

In parallel, improving model robustness to environmental noise remains a critical research direction. Beyond the environmental noises considered in this study, future efforts must prioritize enhancing the model’s discriminative capacity against common environmental interference sources. These include abiotic noises, such as wind-induced branch friction and rainfall impact, and biotic noises from non-target organisms, like bird calls and insect choruses. Collecting and incorporating these typical forest environmental noises into the training datasets will be a critical next step. This process will significantly improve the model’s specificity and reliability, ensuring accurate pest detection in real-world, acoustically complex forest settings.

## 5. Conclusions

This study developed acoustic recognition models for wood-boring pests under different infestation scenarios. In single-species infestation scenarios, three ML models (RF, SVM and ANN) based on feature variables demonstrated perfect accuracy (OA: 100%, Kappa: 1) in identifying target pest species from background noise. However, in co-infestation scenarios with mixed signals, three DL models (AlexNet, ResNet, and VGG) based on time-frequency spectrograms outperformed the ML models. Among them, ResNet achieved the highest OA (88.75% for D1 and 85.0% for D2), effectively reducing the interference from mixed signals.

Future research will focus on several key areas to enhance the practical application of acoustic recognition technology for wood-boring pests. First, the attenuation patterns of feeding vibration signals as they propagate through tree trunks must be explored to optimize sensor positioning and improve detection accuracy. Second, expanding the dataset to include more pest species and host tree types will support the development of a more comprehensive general recognition model. Third, designing a compact, low-power, and IoT-oriented acoustic detection device is crucial. By incorporating edge computing and narrowband wireless networks, the device can minimize the need for high-bandwidth data transmission and tackle the challenges of network coverage and power supply in forest areas.

## Figures and Tables

**Table 1:** Description of seven feature variables.

Variable Type	Variable	Description
Time-domain characteristics	Max amplitude (Max Amp)	The maximum amplitude within the selected sound segment
	Min amplitude (Min Amp)	The minimum amplitude within the selected sound segment
	RMS amplitude (RMS Amp)	The root-mean-square amplitude of the selected part of the signal
Frequency-domain characteristics	Average power density (Avg PD) (dB)	The value of the power spectrum (the power spectral density of a single column of spectrogram values) averaged over the frequency extent of the selection
	Energy (Energy) (dB)	The total energy within the selection
	Peak power density (Peak PD) (dB)	The maximum power in the selection
	Peak frequency (Peak Freq) (kHz)	The frequency at which peak power occurs within the selection

Note: The selection is a 30 ms signal segment.

**Table 2:** Establishment of two independent datasets based on the correspondence between wood-boring pests and host tree species.

Dataset	Host Tree Species	Category	Audio Segments
D1	*P. orientalis*	*S. bifasciatus* feeding signals	100
		*P. aubei* feeding signals	100
		Mixed signals (M1)	100
		Background noises (B1)	100
D2	*F. chinensis*	*A. planipennis* feeding signals	100
		*S. insularis* feeding signals	100
		Mixed signals (M2)	100
		Background noises (B2)	100

**Table 3:** Extraction of audio data from D1/D2 to build subsets of three infestation scenarios.

Infestation Scenarios	Categories Contained in the Subsets	Number of Categories	Audio Segments
Single infestation	Single-pest species feeding signals	*2*	200
	Background noises (B1/B2)		
Co-infestation (without mixed signals)	Two-pest species feeding signals	3	300
	Background noises (B1/B2)		
Co-infestation (with mixed signals)	Two-pest species feeding signals	4	400
	Mixed signals (M1/M2)		
	Background noises (B1/B2)		

## Data Availability

The original data presented in this study are included in the article. Further inquiries can be directed to the corresponding author.
